# The Microstructure Regulation Mechanism and Future Application of Aluminum Alloys Manipulated by Nanocrystalline Structures Formed by In Situ Amorphous Crystallization

**DOI:** 10.3390/ma18174206

**Published:** 2025-09-08

**Authors:** Wen-Bo Yang, Lei Zhan, Lin Liu, Fan-Xu Meng, Run Zhang, Kadiredan Tuerxun, Xing-Rui Zhao, Bai-Xin Dong, Shi-Li Shu, Tian-Shu Liu, Hong-Yu Yang, Feng Qiu, Qi-Chuan Jiang

**Affiliations:** 1 Key Laboratory of Automobile Materials, Ministry of Education and Department of Materials Science and Engineering, Jilin University, Renmin Street No. 5988, Changchun 130025, China; yangwb1622@mails.jlu.edu.cn (W.-B.Y.); 13019105430@163.com (F.-X.M.); mm2665849567@163.com (R.Z.); qdrdt1622@mails.jlu.edu.cn (K.T.); 13734503848@163.com (X.-R.Z.); dongbaixin@jlu.edu.cn (B.-X.D.); tsliu97@suda.edu.cn (T.-S.L.); yanghongyu2021@jlu.edu.cn (H.-Y.Y.); qiufeng@jlu.edu.cn (F.Q.); jqc@jlu.edu.cn (Q.-C.J.); 2 School of Automotive Engineering, Jilin Communications Polytechnic, Changchun 130012, China; 3 Jilin Jiyan New Materials Technology Co., Ltd., Changchun 130025, China; 4 School of Mechanical and Aerospace Engineering, Jilin University, Renmin Street No. 5988, Changchun 130025, China; shushili@jlu.edu.cn; 5 School of Iron and Steel, Soochow University, Suzhou 215021, China

**Keywords:** in situ crystallization, nanocrystal, aluminum alloy, solidification microstructure, microstructure regulation, nanocrystalline nuclei, morphology, structure control, mechanical properties

## Abstract

The present study concentrates on the role and underlying mechanisms of in situ crystallization (employed for nanocrystal formation) in influencing the solidification microstructure and properties of aluminum alloys. By systematically analyzing the effects on α-Al refinement, silicon phase modification, and secondary phase control, as well as exploring the impact on room-temperature mechanical properties, high-temperature deformation behavior, and fatigue performance, this work reveals the potential physical mechanisms of improving mechanical properties by providing nucleation sites and inhibiting grain growth, such as fine-grain strengthening and dispersion strengthening. Moreover, stabilization of the second phase optimizes high-temperature deformation behavior, and a reduction in stress concentration improves fatigue performance. Compared with traditional microstructure control methods, in situ crystallization can achieve deeper grain refinement from micron to nanometer scale, ensuring high uniformity of grain distribution and showing good compatibility with existing processes. By defining the regulation of in situ crystallization on the microstructure and properties of aluminum alloy, the existing research provides a feasible material solution for high stress, high temperature, and high reliability. Its core significance lies in breaking through the performance bottlenecks of traditional modification technology, such as unstable refining effect, element segregation, and so on. The co-promotion of “strength–plasticity–stability” of aluminum alloys and the consideration of process compatibility and cost controllability lay a theoretical and technical foundation for the industrialization of high-performance aluminum alloys.

## 1. Introduction

### 1.1. Aluminum (Al) Alloy

**Figure 1:**
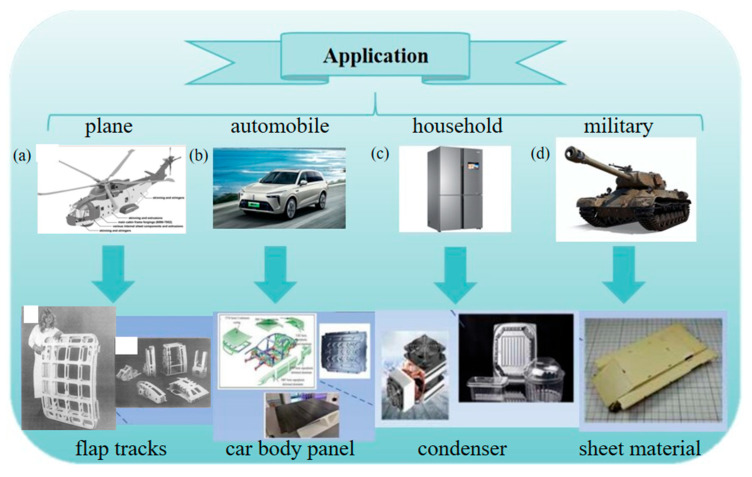
Applications of Al alloys in different fields: (**a**) Al alloy precision casting of baggage compartment door and flap tracks used on Airbus aircraft [6,9]. (**b**) Car body panel used in automobile. (**c**) Condenser used in fridge. (**d**) Sheet material used in tank [10].

As one of the most crucial structural materials in modern industry, aluminum alloys have occupied a pivotal position in aerospace, automotive manufacturing, electronics, and national defense industries because of their low density, high specific strength, excellent corrosion resistance, and superior formability (Figure 1) [1,2,3,4,5]. In the field of aerospace, aluminum alloys have been used in aircraft structures since the 1920s. Aluminum alloys are lighter than other metal materials, and their use reduces the overall mass of aircraft and improves fuel efficiency and payload [6]. In the field of automobile production, more energy-saving vehicles are needed to reduce energy consumption and environmental pollution. Aluminum alloy has the advantages of high specific strength, good formability, and recyclability, and its use is an important way to lighten automobiles [7]. In the field of electronics, efficient conductive materials are the key to achieving efficient energy utilization. Aluminum conductors are not only used in traditional power transmission and distribution, but also widely used in charging stations, positive energy systems, and electric vehicles [8]. According to the alloy composition and application characteristics, aluminum alloys are primarily categorized into Al-Si, Al-Cu, Al-Mg, Al-Zn series, etc. Among them, Al-Si-based alloys, which are characterized by good fluidity and low shrinkage rate, are the most extensively utilized in the industrial field. Currently, they are widely applied in the manufacturing of complex structural components.

However, during the process of manufacturing parts using the casting method, coarse α-Al dendrites, irregular primary Si, and acicular eutectic Si tend to form in aluminum alloys, leading to stress concentration and deteriorated mechanical properties, which restricts their application in high-end fields. Therefore, optimizing the solidification microstructure to enhance performance has become a core research focus in aluminum alloy studies.

### 1.2. Modification and Inoculation of Aluminum Alloy

Moreover, the morphology (including size, distribution, and shape) of primary silicon and eutectic silicon considerably affects the plasticity and fracture behavior of aluminum alloys.

The solidification microstructure of aluminum alloys directly determines their physical properties and service performance. The grain size of α-Al affects the strength and toughness of aluminum alloys. The presence of coarse dendrite structures within the microstructure significantly degrades the fatigue resistance of the material. Moreover, the morphology (including size, distribution, and shape) of primary silicon (Si) and eutectic Si significantly impacts the plasticity and fracture behavior of aluminum alloys [11]. Some traditional microstructure control methods, such as the addition of Al-Ti-B master alloys or natrium (Na)/strontium (Sr) modifiers, can achieve grain refinement of the alloy and improvement of silicon phase morphology to some extent. However, the refinement efficiency is unstable, and issues like element segregation and a narrow processing window are prone to occur. Controlling the coarse primary Si in hypereutectic aluminum alloys with high Si content remains particularly challenging. With the increasing demands for comprehensive properties (e.g., high-temperature strength, fatigue life, reliability) of aluminum alloys in high-end equipment, the development of efficient and precise solidification microstructure control techniques is urgently needed. At present, the main methods for the modification of aluminum alloy are the addition of sodium salt, strontium, rare earth, etc., to the alloy. By restraining the growth of the Si phase, changing the morphology of the impurity phase, or reducing surface tension, the size of the Si phase and grain can be refined, thereby improving the mechanical properties and corrosion resistance of aluminum alloy. Table 1 compares the application effects of different inoculants (Sc, Zr, Sc + Zr, Ti, Nb) in high-strength aluminum alloys prepared by the L-PBF method, covering parameters such as inoculant composition, equiaxed grain area ratio, equiaxed grain size, addition amount, and inoculation methods. Among them, the effect of Sc when used alone is significantly influenced by the addition amount and inoculation method. For example, when 0.66 wt% Sc is added by ball milling, the equiaxed grain area ratio is 60.9% and the size is 2.01 μm, while no effect is observed when 0.7 wt% Sc is added by pre-alloying, and higher addition amounts may improve the effect of pre-alloying. When Zr is added by pre-alloying, the equiaxed grain area ratio ranges from 48.6% to 100%, and the size ranges from 0.38 μm to 0.79 μm; some alloys with high Zr addition can achieve 100% equiaxed grains. The effect of the Sc + Zr composite inoculant is better than that of a single element, with the ball milling method being more effective. For instance, when 1.07 wt% Sc + 0.54 wt% Zr is added, the equiaxed grain area ratio reaches 97.8%. When Ti is added by pre-alloying or vibrative agitation mixing, the equiaxed grain area ratio ranges from 67% to 100%, and the AlTi_2_ alloy has the smallest size of 0.522 μm. When Nb is added by pre-alloying at 1.47 wt%, the equiaxed grain area ratio is 100% and the size is 0.8 μm. Overall, the ball milling method is superior to pre-alloying. Sc and Zr have a synergistic effect when used in combination, and some elements with high addition amounts can achieve fully equiaxed grains, providing a reference for the grain refinement process of aluminum alloys and the selection and parameter optimization of inoculants in L-PBF technology.

### 1.3. Research Background and Significance of In Situ Crystallization for Nanocrystal Formation

The in situ crystallization technique, which induces the formation and controls the growth of nanocrystalline nuclei during the solidification process, provides a novel approach for regulating the solidification microstructure of aluminum alloys, enabling refinement from the microscale to the nanoscale. Grounded in the thermodynamics and kinetics of amorphous crystallization, the technique can generate a high density of nanocrystalline nuclei in supercooled melts by designing alloy composition or external field conditions (such as cooling rate and modification treatment), thereby suppressing excessive grain growth and achieving uniformly fine equiaxed or nanocrystalline structures [21]. The introduction of nanocrystals not only enhances strength through grain boundary strengthening but also improves the distribution of secondary phases and reduces defect initiation.

**Figure 2:**
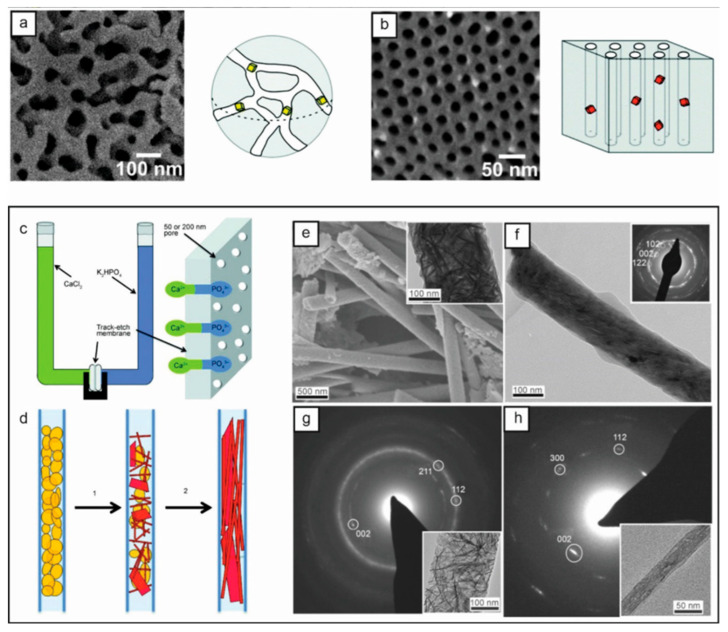
SEM images and diagrams showing glass with controlled apertures: (**a**) 55 nm aperture and (**b**) integral Pt-coated p-PCHE that is 30 nm in diameter with cylindrical aperture array [27]. (**c**) Diagram of calcium phosphate crystallization in a double diffusive configuration in the membrane pore. (**d**) The crystallization sequence’s diagram begins with amorphous calcium phosphate particles, which are yellow; next, they are converted to hydroxyapatite, which is red. Hydroxyapatite’s preferred alignment is the [001] axis aligned with the hole’s long axis. SEM and TEM images (**e**) of crystals grown in 200 nm holes 6 days later and hydroxyapatite rods (**f**) in 50 nm holes 1 day later. Selected area electron diffraction images and relative TEM images of randomly pointed bars (**g**) from a 300 nm aperture and bars (**h**) parallel to a 25 nm aperture [28].

To obtain small nanocrystals, many strategies for synthesizing nanomaterials depend on hard and soft templates to limit size and shape. The porous media on the market, including anodic alumina oxide films, polycarbonate track etching (TE) films, and nanoporous glass, are excellent media for the study of crystallization in confinement. Figure 2 shows a whole platinum-coated p-PCHE of a cylindrical orifice array and a controlled orifice glass. The crystallization mechanism is explained by the crystallization of amorphous calcium phosphate particles to hydroxyapatite.

This paper presents a structured and comprehensive overview of the theoretical foundations, research progress, and mechanisms of in situ crystallization for nanocrystal formation in controlling the solidification microstructure of aluminum alloys. It focuses on the application of this technique in α-Al refinement, silicon phase modification, and secondary phase control; analyzes its enhancement effects on the room-temperature mechanical properties, high-temperature deformation behavior, and fatigue performance of alloys; and outlines future development directions, aiming to provide theoretical references to support the development of aluminum alloys with high performance.

## 2. Theoretical Basis of Formation of Nanocrystalline Structures by In Situ Crystallization

### 2.1. Principle of Thermodynamics and Kinetics of Crystallization of Amorphous Alloys

**Figure 3:**
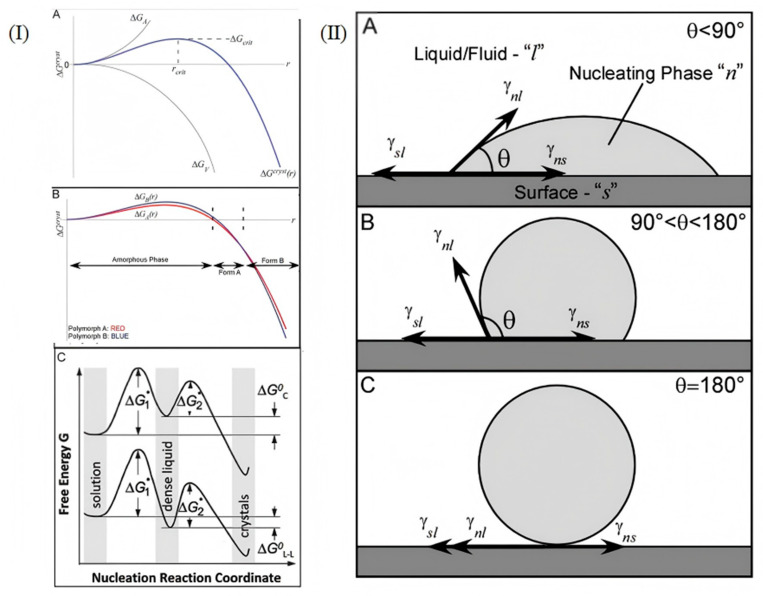
(**I**) (**A**) Functional relationship between the ∆G_cryst_ curve of nucleus growth and the crystal radius r. (**B**) A schematic diagram of the free energy distribution of two competing nuclei of polymorphic forms A and B over a characteristic length range (adapted from [34]). (**C**) A diagram of the two-step nucleation mechanism of crystals, ”*” displays the barrier [35]. (**II**) (**A**–**C**): Static equilibrium diagram in Young’s formula [34].

The formation of an amorphous structure in an alloy or an amorphous alloy is used for the in situ process, which is a result of phase transition from a metastable liquid state to a metastable solid state. Kinetics and thermodynamics are involved in both processes. In the process of nucleation, the basic principle of thermodynamics (solubility, phase diagram) should be used to judge whether nucleation can take place or not, and the kinetics should also be guided. In the process of grain growth, the growth rate can be determined according to surface energy theory [29], two-dimensional nucleation theory [30], and adsorption reaction theory [31]. It is also possible that temperature, solvents, and impurities affect the transformation of crystal patterns and habits. For example, when the interface energy of a grain is high, in order to reduce the surface energy, the grain will reduce the interface area through growth, and the growth rate will be relatively fast. Secondly, the formation of 2D nuclei needs to overcome a certain energy barrier. When the supersaturation of the system is high, the formation of 2D nuclei is favorable. As the frequency of nucleation increases, the growth rate of grains increases. Furthermore, if atoms or molecules are highly adsorbed on the crystal surface and have a high surface diffusivity, they can quickly reach the growth site, promote grain growth, and grow faster. The study of material crystallization theory further explains the influence of thermodynamic and kinetic factors in the crystallization process of materials, thus guiding scientists to develop or improve new growth methods. There are many typical methods for crystallization growth, including gas phase, solution, and melt growth, involving the nucleation of a seed crystal at the growth interface. Undercooling in the melt, supersaturated concentration in the solution, and vapor pressure in the gas phase can be considered as the driving forces for nucleation. But for crystallization confined by nanoscale, the space limitation mainly affects the nucleation process rather than crystal growth [32,33]. Although nucleation is generally regarded as a kinetic process, the properties of confined nanomaterials with size factors approaching critical size are thermodynamic results. When the size of the nucleus reaches a certain level, the body free energy of the nucleus is offset by the unfavorable surface free energy caused by the interface between the nucleus and the growth medium. The Gibbs–Thomson formula predicts the linear relationship between ∆Tm and grain size without changing other parameters. Assuming a contact angle of 180°, this method is suitable for homogeneous cores without infiltrating the hole wall. On this basis, the Gibbs–Thomson equation and Yang equation are combined to obtain the rule of the nucleation phase, the interface energy between the fluids and the matrix, and the melting point. As can be seen in Figure 3(IIA), a good nucleus–surface interaction (i.e., good wettability) yields a result of less than 90°. Figure 3(IIB) Poor nucleus–surface interaction (weak wetting) makes 90° < θ < 180°. Figure 3(IIC) When the Gibbs–Thomson equation is used where θ is generally assumed to be greater than 180°, there is no infiltration at all [21]. The Gibbs free energy analysis in Figure 3 is the key to understanding the thermodynamics of nucleation and growth in amorphous crystallization. For example, in amorphous crystallization of aluminum alloys, competition between surface free energy and volume free energy has to be overcome (Figure 3(IA)), which directly determines the nucleation capacity and stability of nanocrystals. The wetting angle analysis of Figure 3II illustrates the interfacial compatibility between the nanocrystalline nuclei and the aluminum alloy matrix. In aluminum alloys, in situ nanocrystalline crystals need to be well wetted with an α-Al or Si phase (θ < 90°) to effectively nucleate heterogeneous nuclei and refine grain size. According to the Gibbs–Thomson formula, it is necessary to know the interface energy between amorphous and nanocrystalline phases and the driving force of phase change in aluminum alloy. This process can be used to form nanocrystalline materials as well as larger bulk nanocrystalline materials.

### 2.2. Definition, Properties, and Formation Mechanism of Nanocrystals

The core idea of nanocrystalline materials is to introduce a large number of defects into a structure, thus forming a new disordered solid structure in which more than 50% of the atoms (molecules) are contained. To achieve such a high interfacial density, the grain size must be several nanometers (less than 10 nm) [34,35,36,37,38,39]. To this end, Gleiter conducted a systematic study of “nanocrystalline” (also known as “ultrafine crystalline”) material for the first time in Materials Science. Its main components are large-volume-fraction defect nuclei and (stress) lattice regions [40]. Siegel further defined nanocrystalline materials as “polycrystalline materials composed of nanocrystalline grains” in the Annual Review of Materials Science [41].

The key to crystallization of amorphous alloys lies in regulating the nucleation rate as well as the growth rate. By adjusting alloy composition or heat treatment processes (such as isothermal annealing or rapid heating), high-density nucleation and inhibition of grain overgrowth can be achieved, resulting in nanocrystalline microstructures. Nanocrystalline metal at high temperature shows crystal growth [40]. Amorphous alloys undergo glass transition temperature and crystallization temperature during heating. Below the crystallization temperature, the amorphous alloy retains its non-crystalline structure. However, once the temperature reaches or exceeds the crystallization threshold, the amorphous alloy commences the transformation into a nanocrystalline structure.

Crystallization of amorphous alloys is a dynamically controlled process, usually at temperatures well below the melting point of the alloy [42]. The melting point of most aluminum alloys is usually above 600 °C, while the crystallization temperature of amorphous alloys is usually between 200 °C and 500 °C [43]. This is due to the orderly arrangement of atoms in the crystal microstructure; because of the strong adhesion between atoms, the destruction of its lattice requires higher energy. The crystallization process of amorphous atoms is a phase transition from disorder to order; the energy barrier that needs to be overcome is lower than the energy barrier of crystal melting, so the crystallization temperature is far lower than the melting point of crystalline aluminum alloy. Therefore, supercooling exists as the driving force of crystallization.

**Figure 4:**
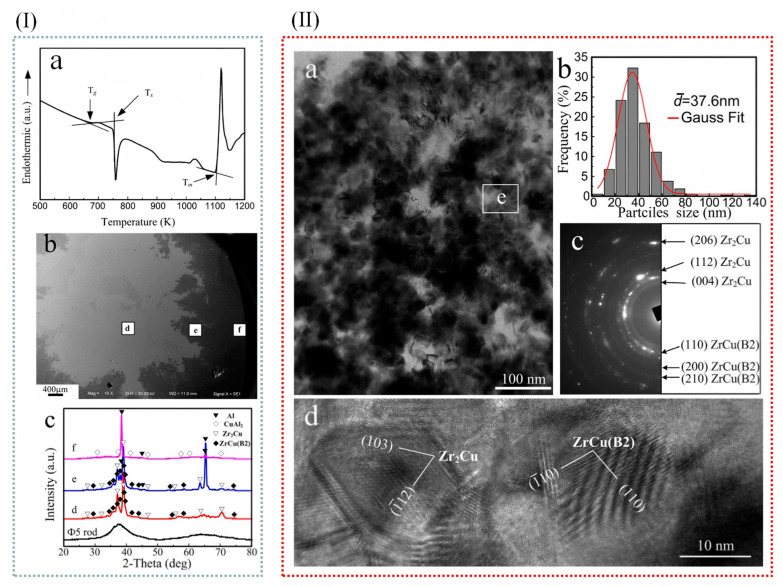
(**I**) (**a**) DSC analysis of the manufactured Zr_55_Cu_30_Al_10_Ni_5_ metallic glass at 20K min^−^^1^ heating rate. (**b**) The macro-profile of the cross-section of the quenched specimen, (**c**) the X-ray diffraction pattern, regions d, e, f of the quenched specimen. (**II**) (**a**) Quenched samples in transition areas were observed by TEM; (**b**) particle size distribution of ZrCu (B2) and Zr_2_Cu particles; (**c**) area e’s selected zone diffraction pattern in (**a**), and (**d**) area e’s typical HRTEM image in (**a**); (**e**) area e (adapted from [44]).

Bai et al. [44] employed differential scanning calorimetry (DSC) to determine the melting point (Tm), crystallization temperature (Tx), and supercooled liquid phase (ΔT = Tg − Tx = 82 K) of amorphous alloys. Whereas the melt pouring temperature of Al-Cu alloy is 1003 K, the amorphous alloy can be crystallized if it is added to the melt of Al-Cu alloy. Figure 4(Ia,b) show the cross-sectional microscopic morphology of a quenched sample. It is evident that in molten aluminum alloys, metallic glass rods dissolve rapidly. Figure 4(Ic) displays the X-ray diffraction (XRD) results of the initial metallic glass strip and three different regions. As indicated, crystallization phenomena were observed within the central region. Specifically, in the d region, diffraction peaks corresponding to ZrCu (B2) and Zr_2_Cu phases were detected. Furthermore, in the e region, the emergence of peaks associated with ZrCu (B2), Zr_2_Cu, and Al phases was noted. Additionally, in the f region, diffraction peaks characteristic of Al and CuAl_2_ phases were identified. The results also show that in the e region, the amorphous alloy dissolves into the Al-Cu melt, and the corresponding α-Al particle size is about 5 nm, while in the f region, the large α-Al particle is dominant, and the average particle size is about 20 nm. Therefore, it can be assumed that Zr_2_Cu and ZrCu (B2) result in grain refinement in the e region. Figure 4(IIa) shows a TEM image of the e region in a quenched sample. Some nanocrystalline particles were also found. Electron diffraction patterns and HRTEM images of the selected areas show that they are ZrCu (B2) and Zr_2_Cu phases. Figure 4(IIb) shows the ZrCu (B2) and Zr_2_Cu particle size distribution as shown in TEM images. It was found that ZrCu (B2) and Zr_2_Cu phases were mainly distributed in the range of 20–60 nm (>85%) with an average particle size of 37.6 nm. For every 10 nm distance, the approximate error is plus or minus 5 nm.

### 2.3. Nanocrystalline Microstructure in Aluminum Matrix Composites

**Figure 5:**
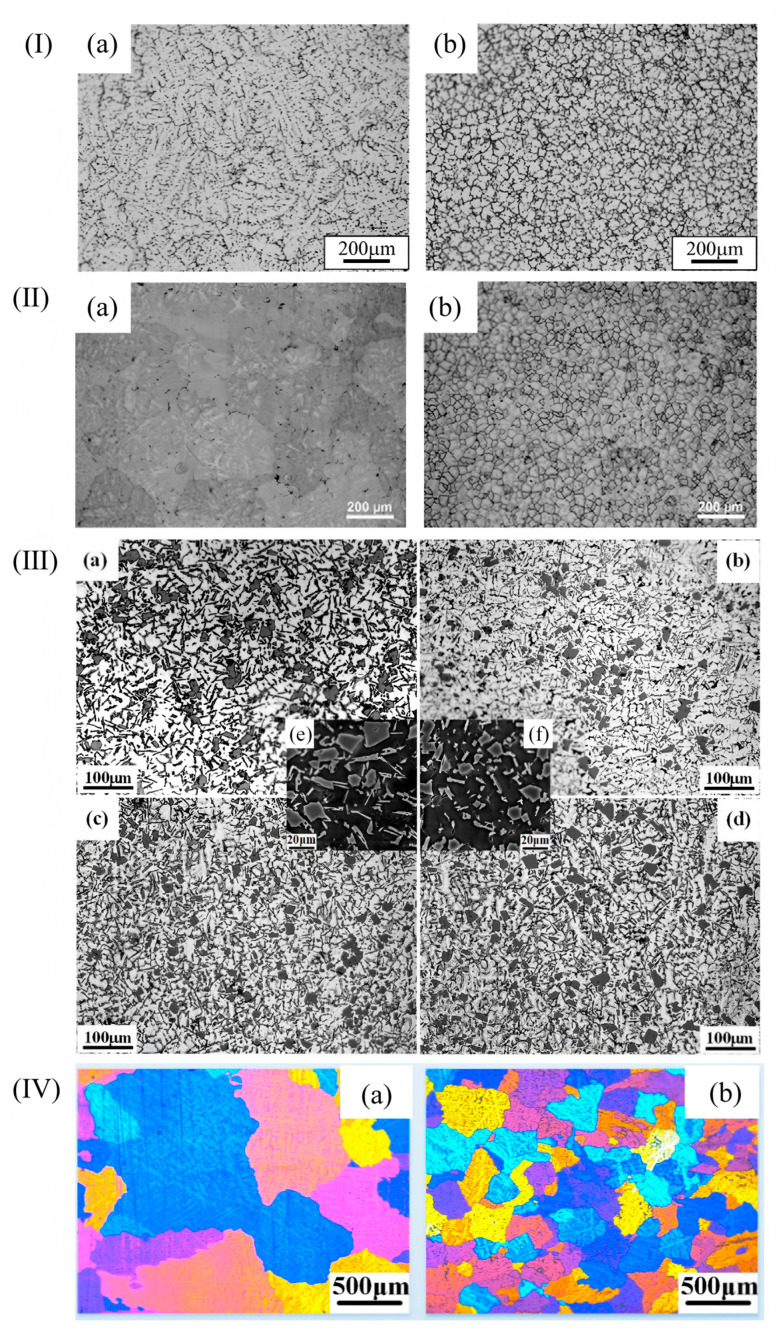
(**I**) (**a**) Uninoculated aluminum alloy; (**b**) inoculated aluminum alloy [45]. (**II**) (**a**) Uninoculated aluminum alloy; (**b**) inoculated aluminum alloy [46]. (**III**) (**a**) Uninoculated aluminum alloy; (**b**) 0.1 wt.% inoculated; (**c**) 0.2 wt.% inoculated; (**d**) 0.4 wt.% inoculated; (**e**) and (**f**) high-magnification SEM images of (**a**) and (**c**) [1]. (**IV**) (**a**) Uninoculated aluminum alloy; (**b**) inoculated aluminum alloy [47].

The common aluminum alloys are Al-Cu alloy, Al-Si alloy, and Al-Mg alloy. For Al-Cu alloys, the microstructure consists of rough α-Al dendrite grains with an average size of about 200 μm. Through the control of nanocrystalline amorphous crystals, α-Al dendritic grains can be refined to tens of microns. For example, FeBSi metallic glasses can produce nano-Al_2_Cu precipitates that refine the α-Al grain size from 200 μm to 42 μm [45]. Zr-based amorphous alloys of 0.2 wt.% reduced it to 35 microns [46]. α-Al dendrites, primary silicon, and eutectic silicon are mainly contained in Al-Si alloys. When 0.2 wt% Zr-based amorphous alloys were added, the size of α-Al dendrites and primary Si decreased from 212.46 μm and 19.24 μm to 143.39 μm and 16.47 μm, respectively. The average size of eutectic Si decreased from 20.21 μm to 11.69 μm [1]. In Al-Mg alloys, the grain size of the matrix of Fe-based amorphous alloys decreases from 1127.6 μm to 238.8 μm [47]. See Figure 5 above.

### 2.4. Application and Development of In Situ Crystallization in Materials Science

As early as 1981, Herbert Gleiter et al. [40] proposed the concept of amorphous nanocrystalline materials. This groundbreaking discovery pioneered research in the field of nanomaterials. Chen, M. W., et al. [45] studied the mechanism of introducing nanocrystalline phases into metallic glasses through amorphization to improve their mechanical properties. Amorphous crystallization can produce uniform nanocrystalline grains, which, as a barrier to dislocation movement, significantly improve material strength and plasticity [48].

The amorphization of nanomaterials has emerged as a burgeoning and pivotal research focus within the scientific community. This process offers a sophisticated means to precisely modulate the microstructural features of nanomaterials, including grain size and spatial distribution, thereby enabling tailored optimization of their physical, chemical, and mechanical attributes. K. et al. [49] prepared transparent conductive oxide thin films through amorphous crystallization. The results demonstrated that amorphization could optimize the grain boundary microstructure and refine the grain size of the thin films, thereby enhancing their electrical conductivity and optical transparency. Herzer, G. [50] successfully synthesized nanocrystalline soft magnetic materials by amorphous crystallization and systematically explored their potential application in electronic devices. The amorphization process was found to be instrumental in optimizing the magnetic properties of these materials, manifesting as enhanced permeability and reduced coercivity, by facilitating the formation of uniformly distributed nanocrystalline grains. But the traditional crystallization theory cannot explain non-classical mechanisms, such as the formation of a mesophase (amorphous, ordered liquid). For example, in in situ growth of metal nanocrystals, dynamic recombination at the atomic level is significantly different from the conventional nucleation–growth model [51]. The uncertainty of this mechanism makes it difficult to precisely control the crystal size, orientation, and phase, especially in nanomaterials. Further research is needed to establish a quantitative relationship model between amorphous composition, melt temperature, cooling rate, and nucleation and growth kinetics.

## 3. Study on Microstructure Control of Cast Aluminum Alloy

Due to their excellent castability, cast aluminum alloys such as the Al-Cu and Al-Si series have garnered significant attention and found widespread applications. Currently, casting technologies including sand casting, permanent mold casting, die casting, and investment casting serve as the primary molding processes for aluminum alloy components. These processes are gradually evolving towards green and intelligent manufacturing. As previously mentioned, Al-Si alloys have emerged as the most extensively utilized aluminum alloys in automotive, aerospace, and equipment manufacturing sectors, accounting for approximately 80% of applications [51,52,53], owing to their superior properties such as low thermal expansion, light weight, good fluidity, excellent weldability, and corrosion resistance [54,55,56,57,58,59,60,61,62,63,64,65,66,67,68,69]. However, the microstructure of Al-Si alloys significantly influences their material properties. For instance, the presence of hard Si phases and acicular eutectic Si phases can substantially degrade the mechanical properties of the alloy [70,71]. Furthermore, primary silicon and coarse α-Al dendrites can induce crack initiation within Al-Si alloys, thereby compromising their fatigue resistance. Through microalloying and the addition of appropriate refiners or modifiers, the microstructure of the alloy can be effectively refined, subsequently enhancing the fatigue resistance of the material [72,73].

### 3.1. Refinement of α-Al and Control of Dendritic Morphology in Cast Aluminum Alloys

The primary mechanism of grain refinement lies in enhancing the nucleation rate while impeding grain growth. This can be achieved by promoting nucleation through heterogeneous nucleation and precise control of the cooling rate. During the solidification process of alloys, an increased number of nuclei leads to the formation of numerous fine grains. The interactions among these grains during growth restrict the grains’ expansion, ultimately resulting in grain refinement. On the other hand, rapid cooling can increase supercooling, which further facilitates the formation of a greater number of nuclei and refines the dendrites. Following this line of approach, a variety of methods for grain refinement in α-Al have emerged, including the addition of grain refiners, sub-rapid or rapid solidification, the incorporation of RE elements, physical treatments, and alloying.

**Figure 6:**
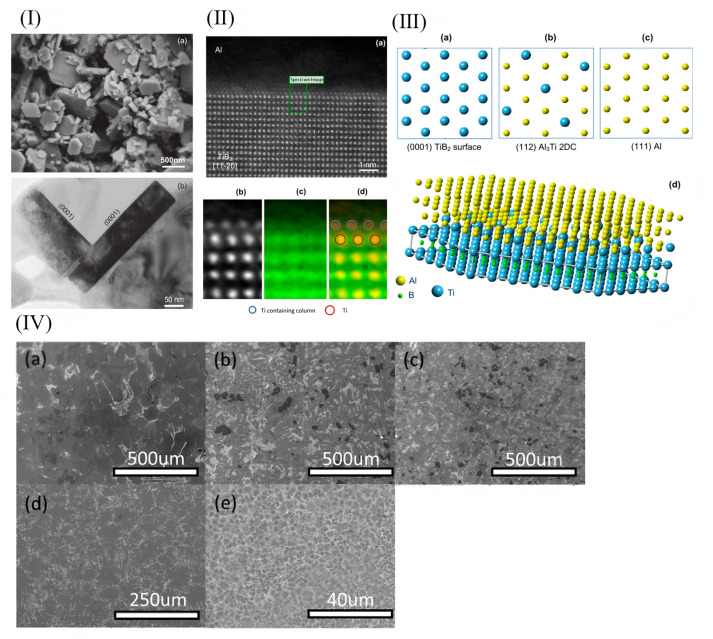
(**I**) (**a**) In a deeply corroded sample of commercial Al-5Ti-1B grain refiners, TiB particles were shown by SEM to have a hexagonal structure; (**b**) TEM images showed that the TiB particles were a typical (0001) crystal plane. (**II**) (**a**) Superparamagnetic Z against HAADF images; (**b**) HAADF with localized Z contrast at the Al/TiB_2_ interface; (**c**) TiK edge (green) atom-resolved EELS images; (**d**) overlap of partial Z contrast images with Ti K edge images. The blue ring represents titanium, and the red ring represents titanium. EELS spectra determined titanium atoms in the monolayer. (**III**) Schematic diagram of heterogeneous nucleation mechanism of aluminum on (0001) surface of TiB_2_ particles with (112) Al_3_Ti 2DC single layer. Atomic match at TiB_2_/Al_3_Ti 2DC/Al interface. (**a**) TiB_2_ plane; (**b**) Al_3_Ti 2DC plane; (**c**) Al plane; (**d**) Atomic matching [77]. (**IV**) The AlSi_13_Cu_4_MgNi_2_ alloys solidified with different cooling rates are analyzed by SEM (°C/s): (**a**) 0.15; (**b**) 1.5; (**c**) 15; (**d**) 150; (**e**) 1.5 × 105 [78].

The addition of grain refiners to aluminum alloy melts during the solidification process has been demonstrated to not only effectively achieve grain refinement but also improve the solidification microstructure and mechanical properties of the alloys. Currently, refiners of the Al-Ti-C-B, Al-Ti-B, and Al-Ti-C systems are commonly employed for grain refinement in aluminum alloys, with the Al-Ti-B system being relatively more widely utilized. The Al-Ti-B system additives are typically prepared using the “trial-and-error method” [74,75,76]. However, the exact mechanism by which the Al-Ti-B system refines the grains of aluminum alloys remains unclear. Relevant research findings indicate that TiB_2_ particles coated by a Al_3_Ti two-dimensional compound (2DC) can effectively promote heterogeneous nucleation, whereas those not encapsulated by Al_3_Ti do not contribute to heterogeneous nucleation. Fan et al. [77] demonstrated the presence of a Al_3_Ti two-dimensional compound (2DC) thin film on (0001) TiB_2_, which can enhance the properties of TiB_2_ particles. When the Al-Ti concentration in the melt is relatively high, Al_3_Ti(2DC) forms at the interface between Al and TiB_2_. The formation of Al_3_Ti (2DC) and the sufficient amount of free Ti in the melt synergistically enhance the ability of TiB_2_ grains to transform from columnar to equiaxed morphology. Consequently, the alloy grains are refined, as shown in Figure 6. However, it should be noted that TiB_2_ particles can easily agglomerate during the melting process, which will split the matrix and destroy the uniformity of the microstructure. Secondly, the Ti element in Al-Ti-B master alloy can easily be replaced with Zr, Cr, V, and other trace elements in aluminum alloy, thus weakening the effect of grain refinement. And with the extension of incubation time, the refinement effect will gradually weaken; this is the phenomenon of refinement “decline”.

The solidification rate also significantly influences the microstructure of aluminum alloys, primarily because different cooling rates provide varying degrees of undercooling, altering the kinetics of dendrite formation and thus resulting in microstructures with distinct grain sizes. Tian et al. [78] studied the microstructure and mechanical properties of Al-13Si-4Cu-1Mg-2Ni alloy at different cooling rates. It was found that the second branch arm spacing decreases significantly with an increase in cooling rate. This is mainly because the critical nucleation radius and activation energy of nucleation decrease and the nucleation rate increases with an increase in cooling rate. Rapid cooling makes the front of the solid–liquid interface have a high temperature gradient, which promotes the heterogeneous nucleation of Al grains in the matrix and inhibits their growth. Under the conditions of sub-fast and fast solidification, the solidification speed and composition play a decisive role in the microstructure of materials. Under different cooling rates and different degrees of supercooling, the dynamics of the solidification process will change, so the size and morphology of dendrites will change. But this method is only suitable for small-batch production.

**Figure 7:**
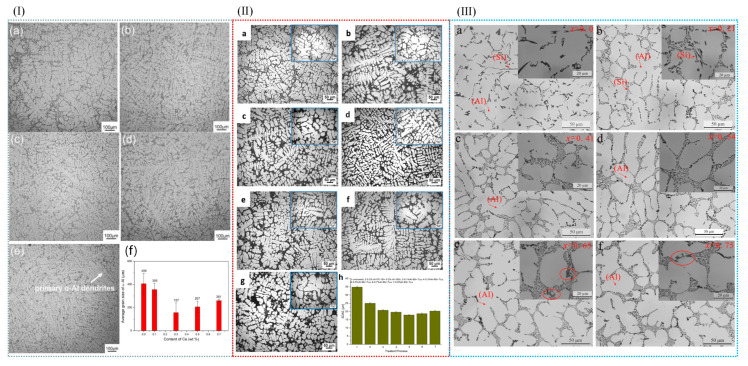
(**I**) The microstructure of A356 alloy with different cerium contents was analyzed by optical microscope. (**a**) A356 (**b**) A356 + 0.1% Ce by weight; (**c**) A356 + Ce (weight) 0.3%; (**d**) A356 + Ce (weight) 0.5%; (**e**) Ce (weight) A356 + 0.7%; (**f**) average particle size of a-Al with different cerium contents in a-Al [79]. (**II**) Microstructure evolution of A356.2 alloy treated with different processes: (**a**) untreated, (**b**–**f**) Al-6Sr-7La (0.1%, 0.3%, 0.5%, 0.7%, 0.9%), (**g**) prepared from 0.2% Al-5Ti-1B and 0.2% Al-10Sr master alloy. (**h**) SDAS evolution of A356.2 alloy in different processes [80]. (**III**) Microscopic images of A356-XSC alloy in different casting states obtained by low- and high-magnification microscope: (**a**) A356 alloy; (**b**) A356-0.21Sc; (**c**) A356-0.41Sc; (**d**) A356-0.54Sc; (**e**) A356-0.65Sc; (**f**) A356-0.75Sc [81].

In addition to the aforementioned methods, the addition of RE elements to aluminum alloys can also refine the grain size of α-Al. Niu et al. [79] investigated the effect of cerium (Ce) on the microstructure of A356 alloy. The results revealed that the addition of Ce significantly refined the grain size of A356 alloy, with the optimal grain refinement achieved at a Ce content of 0.3 wt.% (see Figure 7I). Owing to the grain refinement, the castability of A356 alloy was also significantly improved. Qiu et al. [80] conducted an investigation into the effects of La and Sr on A356 alloy. Their findings demonstrated that when 0.5 wt% Al-6Sr-7La master alloy was added, the secondary dendrite arm spacing (SDAS) was reduced to 17.9 µm, and the original acicular eutectic silicon transformed into a fibrous morphology, as depicted in Figure 7II. In the A356 alloy system, when the Sc content reached 0.54%, the elevated Si content in the melt constrained the growth rate of the primary α-Al phase, resulting in a finer primary phase structure. This silicon-rich environment increased the supersaturation degree of Si in the melt, thereby enhancing its nucleation efficiency and further modifying the morphology of the primary α-Al phase [81], as illustrated in Figure 7III. The addition of RE elements makes the solute content and the distribution of solute in the front of the solidification interface change; thus, the grain size of the alloy becomes smaller. RE elements have the advantages of lowering the initial crystallization temperature, micro solid solution in the alloy phase interface, inhibiting grain growth, and large undercooling.

**Figure 8:**
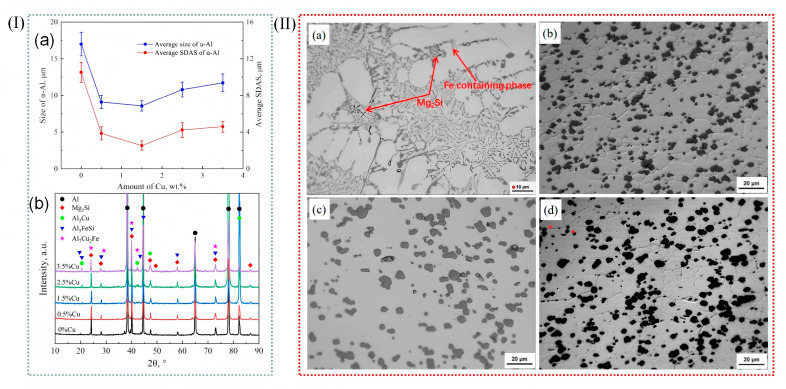
(**I**) (**a**) Average grain size (Lα) and secondary dendrite arm spacing (SDAS) of the primary α-Al phase in Al-10Mg_2_Si alloys with different Cu contents. (**b**) XRD patterns of Al-10Mg_2_Si alloys with different Cu contents [83]. (**II**) Optical micrographs under different states: (**a**) as-cast; (**b**) T1; (**c**) T4; (**d**) T6 [84].

Wu et al. [82] introduced trace amounts of copper into Al-10Mg_2_Si alloy, which effectively refined the primary α-Al dendrites. The results indicated that while the average length of the primary α-Al dendrites was not directly stated to increase, the secondary dendrite arm spacing was significantly reduced by 352.2%, and the length of the primary α-Al dendrites was decreased by 97.7%, indicating a dramatic refinement effect. Various alloying additions, as well as processing techniques such as extrusion, rolling, laser processing, and friction stir welding, exert a profound influence on the microstructure of aluminum alloys [83], as exemplified in Figure 8I. Wang et al. [84] analyzed the microstructural evolution of Al-Si-Mg alloys in different states during friction stir welding. Their study revealed that the microstructure of the nugget zone was largely independent of the initial state of the matrix material, as shown in Figure 8II.

### 3.2. Modification and Refinement of Primary Silicon in Cast Aluminum Alloys

**Figure 9:**
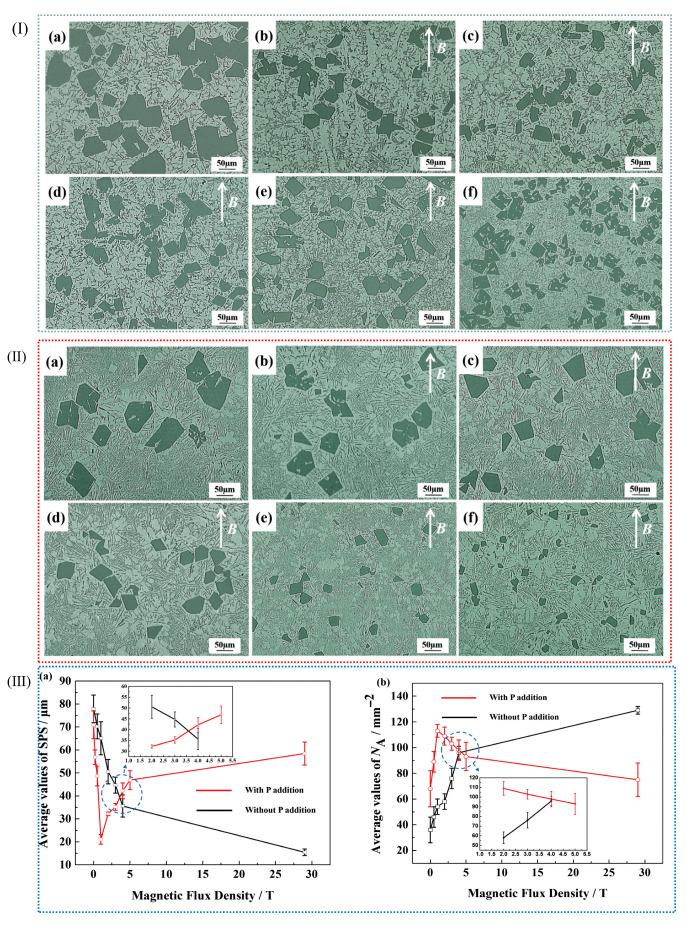
(**I**) Micrographs of Al-20 W% Si alloy containing P at different B levels: (**a**–**f**) 0 T, 0.5 T, 1 T, 3 T, 4 T, 29 T. (**II**) Micrographs of Al-20 Wt.% Si alloy without P addition at different B levels: (**a**–**f**) 0 T, 0.5 T, 1 T, 3 T, 4 T, 29 T. (**III**) In Al-20 Wt.% Si lines, the average SPS added by P is (**a**) SPS, and the average NA content is (**b**) NA [90].

The mechanical properties of aluminum alloys depend on their microstructure morphology, including the sizes and morphology of α-Al, eutectic Si, primary Si, and the second phase [79,80,81,82,83,84,85,86,87,88]. The refinement of primary silicon in eutectic Al-Si alloys is usually achieved through methods such as chemical addition, thermal control, and dynamic grain refinement. Among them, the chemical addition method is the most commonly used in industrial applications because of its simple operation and remarkable refinement effect. It was shown that P, Sr, Na, and RE elements can refine the primary silicon in Al-Si alloy obviously [89,90]. P (e.g., Si-P, Cu-P, Al-Cu-P, Al-Fe-P) is added to hypereutectic Al-Si alloy to form an AlP phase (Al [l] + P [l] → AlP [s]), promoting the nucleation of primary silicon through AlP and further refining primary silicon, as shown in Figure 9 [90].

**Figure 10:**
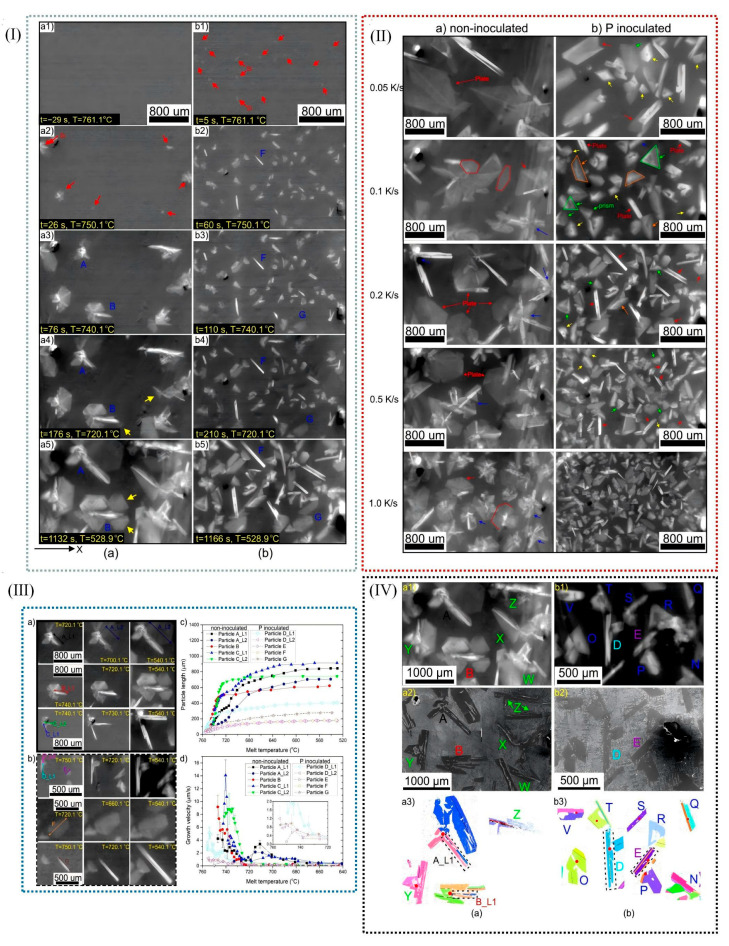
(**I**) (**a**) Field study X-ray images of uninoculated Al-22Si-18.8Cu. (**a1**–**a5**) Uninoculated microstructure in different time and temperature noted in the picture and (**b**) Al-22Si-18.8Cu alloy inoculated with 110 ppm P cured at the same cooling rate. (**b1**–**b5**) Inoculated microstructure in different time and temperature noted in the picture. (**II**) During the solidification process of (**a**) the uninoculated alloy and (**b**) the alloy inoculated with 110 ppm P, recorded by X-ray imaging techniques, the morphology of initial silicon grains of Al-22Si-18.8 Cu series alloys at different cooling rates under FOV conditions was studied. (**III**) (**a**,**b**) Evolution of the size and morphology of the main silicon grains selected during curing of the above two alloys; (**c**) evolution of the size of the single branch and flat plate of porous silicon grains marked by (**a**,**b**); the measurement error of the instrument is ±4.5 mm. (**d**) The growth rate of a silicon particle is calculated from the growth curve expressed in (**c**). (**IV**) The primary silicon particles of the two alloys during solidification were observed in real time by X-ray, and their microstructures were also observed. (**a1**) non-inoculated and (**b1**) P inoculated alloy. The post-solidification structure in (**a2**,**b2**). EBSD directional diagrams of certain raw silicon particles (**a3**,**b3**) [91].

During their investigation of the growth kinetics of primary silicon particles (PSPs), Xu et al. [91] discovered that the addition of P could effectively reduce the growth rate of primary silicon. With the incorporation of P, the morphology of silicon underwent a transformation from star-shaped and interconnected multilayer structures to dense, massive, and single-chip configurations. Crystallographic studies on primary silicon revealed that, in the absence of P, plate-like PSPs in aluminum alloys grew through a biplanar depression mechanism. However, following P addition, the massive PSPs in aluminum alloys did not exhibit growth via this biplanar depression mechanism (as illustrated in Figure 10). A comparison between the simulated and measured growth results of primary silicon demonstrated that the growth kinetics of PSPs closely approximated diffusion-controlled kinetics under high undercooling conditions.

R. Haghayeghi [92] analyzed the effect of Sr and Sb on primary Si in A390 hypereutectic aluminum–silicon alloys and proposed an enhanced impurity-induced twinning (IIT) theory for the evolution of primary silicon, where twins, stacking faults, and kinks help control the growth of silicon. Sb refines primary silicon crystals through twining and increasing the density of stacking faults. Conversely, the presence of Sr enhances the entanglement effect, promoting the refinement of primary Si. Vijeesh Vijayan et al. [93] investigated the effects of combined modification with strontium (Sr) and nickel (Ni) on the mechanical properties and microstructure of hypereutectic Al-23Si alloys. Both Ni and Sr elements cause the star-shaped primary Si to transform into polyhedral crystals and refine these crystals. However, when Sr and Ni are employed in combination for the aluminum alloy modification, Sr is found to attenuate the grain refinement effect of Ni within the alloy. This interaction results in the formation of primary silicon and triggers a “poisoning reaction,” which subsequently degrades the mechanical properties of the material. In addition to the incorporation of alloying elements, rapid solidification techniques can also be utilized to enhance the solubility of Si in the Al melt (increasing it from 1.65% to 10–16%). This process effectively suppresses the nucleation and growth of Si, thereby refining its microstructure.

### 3.3. Modification and Control of Eutectic Silicon in Cast Aluminum Alloys

Eutectic silicon usually exists in aluminum alloy in the form of sheets or needles. This form of eutectic silicon is prone to stress concentration and crack initiation. When the stress reaches a certain degree, cracks will occur at the interface of eutectic Si and Al matrix or inside eutectic Si and expand rapidly, resulting in a decrease in toughness and plasticity. Therefore, it is very important to control the size and morphology of the eutectic Si phase in Al-Si alloy to meet the requirements of automotive and aerospace applications [94,95,96]. Researchers worldwide have conducted extensive investigations into the modifiers employed for aluminum and its alloys, leading to the development of a range of effective modifiers tailored specifically for aluminum–silicon alloys. Notable among these modifiers are Na, Sr, and RE elements. Several commonly encountered challenges or “spoilers” associated with the modification process are discussed below. Table 2 displays the modification of eutectic Si by different metamorphic elements.

Na modification is one of the commonly employed processes in aluminum alloy production, primarily aimed at improving the morphology and distribution of silicon phases within the alloy. Due to its highly reactive nature, Na is typically not added in its metallic form but rather as sodium compounds, specifically Na salts. Commonly utilized Na salts in industrial applications include NaF, NaCl, Na_3_AlF_6_, etc. The addition of these Na salts for modifying aluminum alloys offers several advantages, such as effective modification, insensitivity to the cooling rate of castings, and the absence of a latent period. Research conducted by Li et al. [97] has demonstrated that the introduction of Na positively influences the diffusion between eutectic silicon and aluminum, thereby inhibiting the growth of eutectic silicon. Moreover, Na addition also affects the twin density within the alloy. When the Na content reaches 160 ppm, the twin density in the Al-5Si alloy is measured at 4.8 ± 0.85 × 10^−2^ nm^−1^, which is two orders of magnitude higher than that in the unmodified Al-5Si alloy. This phenomenon is primarily attributed to the propensity of Na to adsorb onto silicon, promoting the formation of silicon twins. It should be noted, however, that the application of Na salt modifiers is constrained by issues such as volatility, oxidation, low recovery rates, and difficulties in addition and regulation.

Strontium (Sr) represents another effective modifier that is commonly introduced into aluminum alloys through the addition of master alloys. The Sr-containing master alloy is added to the aluminum alloy melt under specific temperature and time conditions, followed by homogenization to achieve the desired modification effect. A study conducted by Gan et al. [54] revealed that in an unmodified Al-7Si alloy, the eutectic Si phase exhibited a coarse α-Al_2_O_3_-like structure, with a maximum particle size reaching 20–30 µm. However, upon modification with Sr, the eutectic Si transformed into a fine fibrous parallel distribution, with the diameter reduced to 0.2–0.3 µm and the tips assuming a nearly hemispherical shape, as illustrated in Figure 10I. The mechanism of Sr ion modification has been the focus of attention [98,99,100,101]. The two most commonly used modification methods are inhibition of impurity-induced twinning (IIT) and TPRE [54]. The TPRE [101] mechanism refers to the formation of twin valleys at the crystal front of the Si wafer during eutectic growth. Eutectic Si grows along the preferred direction in (111) Si [211] Si. Modifiers can easily adsorb and concentrate in the valley of twins, hindering the further diffusion of Si atoms to the valley, inhibiting the growth of Si, and changing the direction of Si growth, causing Si to grow in the direction of [100] and [110]; this forces it to produce more branches and changes the morphology from sheet to fiber. IIT refers to the phenomenon of forming twin structures in crystal materials due to impurities, such as trace alloy elements, doped atoms, or foreign contaminants. The twins induced by the concave edge of the twin plane can be rapidly nucleated by means of stress concentration with good coordination and controllability with the substrate, but they depend on the special geometric structure and are restricted by the direction of load; as a result, the anisotropy can easily be intensified. IIT has a wide nucleation range, strong applicability, and a stable strengthening effect and can be controlled by composition, but interface defects, which are sensitive to impurity content and prone to decreasing plasticity, may be introduced. In practice, the material properties are often optimized according to their advantages [102].

**Figure 11:**
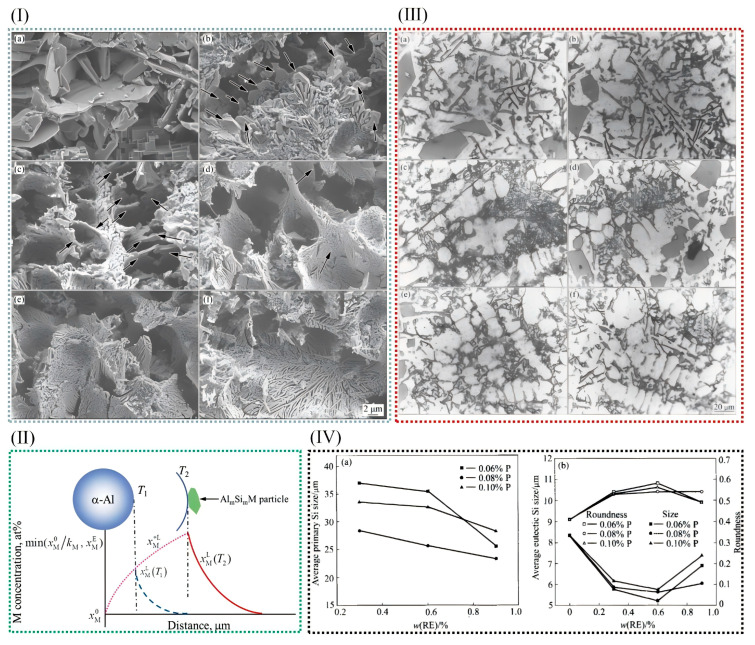
(**I**) Scanning electron microscope images of eutectic Si phases in unmodified Al−7Si alloy (**a**) and (**b**–**f**) Sr-modified Al−xSi alloys (x = 3, 5, 7, 9, and 12) [54]. (**II**) There is a concentration distribution of M in front of α-Al particles [103]. (**III**) Al-2Si alloys’ eutectic silicon morphologies modified with different modifiers: (**a**) P + RE-free; (**b**) P 0.06% + RE 0.3%; (**c**) P 0.06% + RE 0.6%; (**d**) P 0.06% + RE 0.9%; (**e**) P 0.08% + RE 0.6%; (**f**) P 0.10% + RE 0.6%. (**IV**) The effect of RE elements on the microstructure of primary and eutectic silicon in Al 20% alloy: (**a**) primary Si; (**b**) eutectic Si [104].

In addition to the aforementioned elements such as Na and Sr, RE elements, including cerium (Ce), lanthanum (La), and yttrium (Y), have also been employed for the modification of aluminum alloys. These rare earth elements possess unique electronic structures and chemical properties. They can interact with other elements within an aluminum alloy, thereby improving the performance of the aluminum alloy. On the other hand, they can be used as a heterogeneous nucleation core, increasing the number of nucleation sites during the solidification process of the alloy and thus refining the grain structure. Zhang et al. [103] investigated the modification effects of elements including Na, Sr, Eu, Yb, etc., on hypoeutectic Al-Si alloys. They found that the solubility of the modifying elements (Ms) (Na, Sr, Eu, Yb) in the α-Al system and the interaction energy parameters between these elements and the alloy matrix were crucial factors influencing the modification efficacy. The solubility of Ms in the α-Al system is low, and the interaction between Ms and Si (or Al) is weak, making these elements have a good modification effect (Figure 11II). Chen et al. [104] modified aluminum peroxide–silicon alloy with phosphorus and RE elements. Their research findings indicated that the addition of RE elements had a great influence on the microstructure of the materials. The combined P and RE element additives remarkably refined the massive primary silicon and transformed coarse needle-shaped silicon into fine fibrous and lamellar eutectic structures. Although the P element can promote the formation of primary silicon, an excessive amount of P was harmful to the refinement of primary silicon. In contrast, RE elements demonstrated a favorable refinement effect on both eutectic silicon and primary silicon (as illustrated in Figure 11III,IV).

Therefore, Na, Sr, Ba, Sb, and RE elements are efficient eutectic silicon modifiers that can change the shape of eutectic silicon from needle to fiber, sheet, or layer. However, the above modifier elements can only refine the eutectic Si in Al-Si alloys, but not the α-Al phase.

### 3.4. Compound Modification Control in Cast Aluminum Alloy

**Figure 12:**
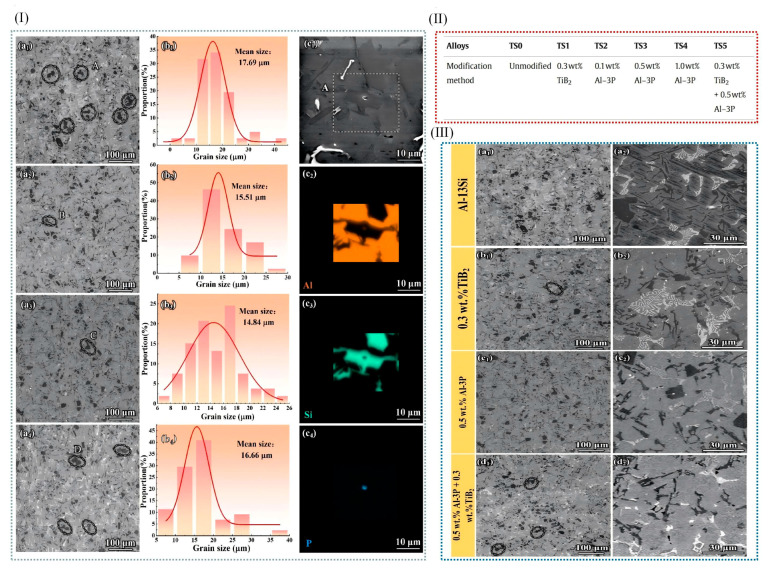
(**I**) Inoculated Al–13Si alloy’s microstructures: (**a1**–**a4**) TSx (x = 0, 2, 3, 4). Primary silicon phases’ size statistics: (**b1**–**b4**) TSx (x = 0, 2, 3, 4). (**c1**) TS3’s images in BSE; (**c2**–**c4**) area A in EDS: (**c2**) Al; (**c3**) Si; (**c4**) P element. (**II**) Modification modes for relatively numbered alloys. Containing different components of TiB_2_ and Al-3P. (**III**) SEM images: (**a**–**d**) low (**a1**–**d1**) and high (**a2**–**d2**) magnification SEM photographs of TSx (x = 0, 1, 3, 5) [11].

The grain refinement mechanisms vary among different modifying elements. Elements such as Na and Sr can form a film on the surface of the Si phase, thereby inhibiting the growth of silicon and achieving a refinement effect. The addition of RE elements provides additional nucleation sites during solidification. Moreover, RE elements react with impurities in aluminum alloys to form high-melting-point compounds, further enhancing the nucleation rate and achieving grain refinement. The refinement effect achieved by adding a single-phase element is limited. However, the synergistic effect of different refinement mechanisms can be realized through the combined use of multiple modifiers, leading to a more refined, homogeneous, and dense alloy microstructure [105]. Li et al. [6] investigated the effects of adding P and TiB_2_ on Al-Si alloys. The results demonstrated that the addition of P alone could refine primary silicon, while the addition of TiB_2_ alone could refine α-Al dendrites and eutectic silicon [72,106]. When a P-TiB_2_ composite additive was introduced, both the Si and α-Al phases in the Al-Si alloy were refined. Consequently, the mechanical properties of the material, particularly its high-temperature mechanical properties, were significantly improved. Compared with unmodified Al-13 Si alloy, Al-3P/Al-13 Si 0.5 wt% + TiB_2_ 0.3 wt% alloy increased by 36.4%, 31.7%, and 32.1% at 250 °C and 60.0%, 42.0%, and 50.9% at 300 °C (Figure 12). When only P was added, the AlP phase in Al-3P was used as a heterogeneous nucleation nucleus of the primary silicon phase, and the refining of the primary silicon phase was best at 0.5 wt% by reducing its nucleation supercooling and increasing its nucleation rate. TiB_2_ nanoparticles provided high-density nucleation sites for the α-Al phase, and further refined it from large dendrites to equiaxed crystals; at the same time, they limited the growth region of the eutectic silicon phase to refine the eutectic silicon phase, and they refined the precipitated phase because of the dislocation caused by the difference in thermal expansion coefficient with the Al matrix. In the P-TiB_2_ complex, Al-3P and TiB_2_ played their respective refining roles, and AlP could easily form a coupling compound on the TiB_2_ surface, further refining it as a heterogeneous nucleation point of the primary silicon phase. It reduced coplanar silicon at the same time, refining it into the main feature of eutectic silicon phase and refining it more remarkably, and it reduced cocrystallization temperature to promote cocrystalline transition.

In the last twenty years, Al-Ti-B grain refiners have been widely used, but many problems have been found in their application. Murty et al. [107] found that the refinement effect of Al-Ti-B master alloys is not good because of the poisoning effect of Cr, Zr, Si, and Li. In addition, Schaffer systematically investigated the precipitation process of Al-Ti-B master alloy particles and verified that there are a lot of insoluble TiB_2_ particles in Al-Ti-B master alloy, which can easily agglomerate [108]. Comparatively, TiC produced from the Al-Ti-C master alloy can agglomerate more easily than TiB_2_ particles, while its wettability with molten metal is not good, which makes the alloy preparation difficult [109]. At the same time, Vandyoussefi et al. [110] investigated the addition of Al-Ti-C particles to Al and Mg alloys. Their findings revealed that the Al-Ti-C grain refiner exhibited significant fading behavior with prolonged holding times, attributed to its instability in the molten state. Much work has been carried out to address these issues for Al-Ti-B and Al-Ti-C master alloys, such as the optimization of the preparation process [111,112,113,114] and the addition technology and the introduction of new carbon sources [109].

**Figure 13:**
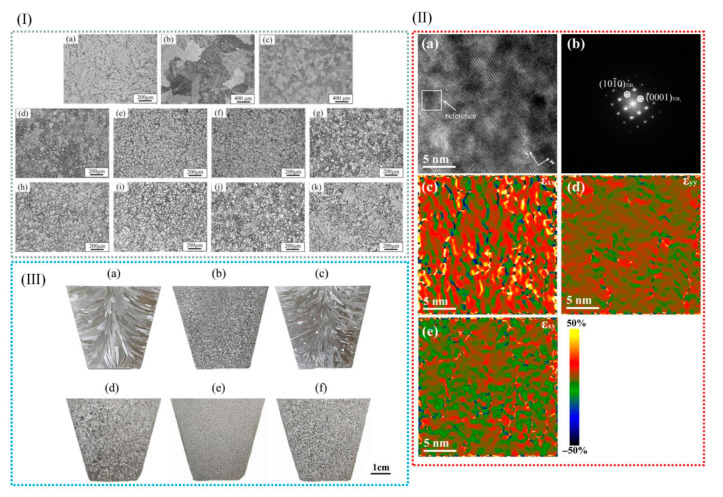
(**I**) Microstructures of (**a**) uninoculated alloy; (**b**) Al–Cu alloys that are uninoculated (no Ti and Zr); and (**c**) Al–Cu alloys that are inoculated (no Ti and Zr) with FeBSi 0.1 wt.% metallic glass for 5 min. (**d**–**k**) Microstructures of inoculated Al–Cu alloys in which different metallic glass contents are used with different inoculation times: (**d**–**g**) 5 min: (**d**) 0.05 wt.%, (**e**) 0.1 wt.%, (**f**) 0.2 wt.%, (**g**) 0.4 wt.%; (**h**) 1 min, 0.1 wt.%; (**i**) 10 min, 0.1 wt.%; (**j**) 30 min, 0.1 wt.%; and (**k**) 60 min, 0.1 wt.% [45]. (**II**) (**a**) Lattice images of TiB_2_ in the direction of [010] from high-resolution transmission electron microscopy, in which white squares represent reference regions; (**b**) power spectra of high-resolution transmission electron microscopy imaging and resolution points; and (**c**–**e**) strain fields of εxx, εyy, and εxy. (**III**) (**a**–**f**) The toxicity of Sc, i.e., Al-5Ti-1B’s Sc eliminating fine-grained effects, is clearly shown [83].

Bai [45] reported that the addition of 0.1 wt% FeBSi can result in an obvious refining effect for Al alloy with a 42–200 μm grain size range. The Fe_2_B phase was identified as crystalline at the heterogeneous nucleation sites of the α-Al particles (Figure 13I). The results showed that during a 30 min incubation period, the microstructure of the Al-Cu alloy was optimized, leading to enhanced strength and plasticity. Huang et al. [83] used HRTEM to investigate the diffusion behavior of Sc in TiB_2_ (0001) crystals, and they utilized geometric phase analysis (GPA) to visualize stress concentration, thereby elucidating the poisoning mechanism of Sc (Figure 13). Their findings (Figure 13(IIIa–f)) clearly demonstrated the detrimental effect of Sc on the Al-5Ti-1B refiner, specifically its role in weakening grain refinement. This study pioneered the investigation of Sc-induced toxicity in Al-5Ti-1B refiners and highlighted the necessity for caution when selecting Sc-containing grain refiners to avoid compromising refining efficiency [83]. When multiple grain refiners and modifiers are used together, there is an interaction or poisoning effect between the grain refiners and modifiers, which weakens the grain refining effect. Therefore, it is of great significance to develop a green, low-cost, high-refining-efficiency, multi-component co-control cast aluminum alloy microstructure regulator.

### 3.5. Control of the Second Phase in Cast Aluminum Alloy

There are many kinds of second phases in aluminum alloys, including Si, Cu, Mg, Zn, and iron impurities contained in small amounts of silicon and raw materials (such as aluminum ingots, scrap aluminum, etc.) brought in by furnace charge and flux during melting. After proper heat treatment, precipitated phases can be produced, which can significantly improve the room-temperature and high-temperature mechanical properties, creep resistance, and fatigue resistance of the material. The microstructure of an Al-Si series alloy consists of an α-Al matrix, primary silicon, and a nano/microscale flaky or fibrous Si eutectic mixture. An Al-Cu series alloy is mainly composed of an α-Al matrix and the CuAl phase. During aging, different metastable phases such as the GP, θ″ phase, and θ′ phase are formed. An Al-Mg series alloy is mainly distributed on the grain boundary and is composed of an α-Al matrix, Mg_5_Al_8_, and Mg_2_Si (if the silicon content is high), which can hinder dislocation movement. In an Al-Zn series alloy, the MgZn phase and other related phases are distributed in an α-Al matrix, and the GP region, η″ phase, and η′ phase are metastable during aging. This ordered and fine microstructure greatly improves the strength of aluminum alloy. However, the increase in strength of aluminum alloy is usually accompanied by a decrease in plasticity, while the intermetallic compounds are brittle. By refining the microstructure of aluminum alloy, the strength of aluminum alloy can be improved effectively under the premise of ensuring its plasticity.

**Figure 14:**
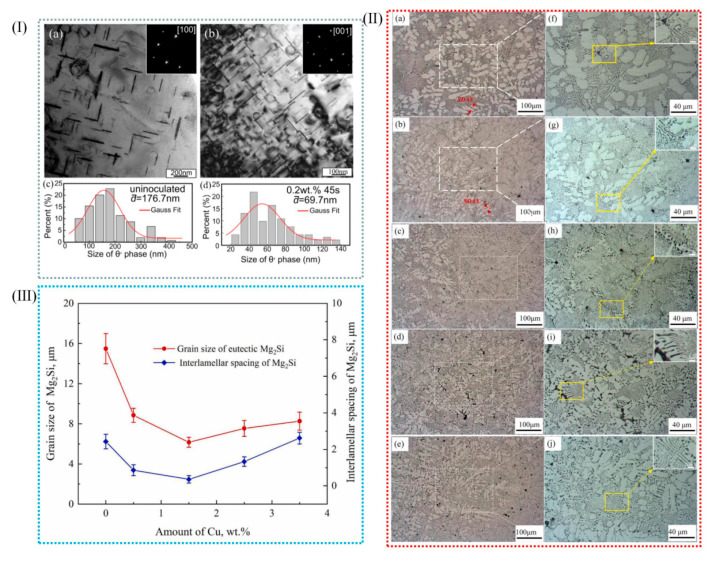
(**I**) (**a**) TEM photographs of uninoculated alloys and (**b**) TEM images of inoculated 0.2 wt% precipitated theta phases (**c**,**d**) relative to the statistical results of the size of the precipitated phase [46]. (**II**) The as-cast AlMg_10_Si_2_ alloys’ OM images with different Cu contents (wt%): (**a**,**f**) 0.0, (**b**,**g**) 0.5, (**c**,**h**) 1.5, (**d**,**i**) 2.5, and (**e**,**j**) 3.5. (**III**) The eutectic Mg2Si phase’s average grain size (LMg_2_Si) and interlamellar spacing (ILS) in the Al-10Mg2Si alloys with different Cu proportions [82].

Al-Cu alloys have high specific strength and good fatigue resistance, thermal conductivity, electrical conductivity, electromagnetic shielding, processing performance, etc. In addition to being strongly dependent on grain size, the strength and ductility of materials depend on the precipitation hardening of aging aluminum alloys to achieve both high strength and ductility [115,116]. Bai et al. [46] have successfully prepared in situ Zr-based metallic-glass-reinforced Al-Cu matrix composites by using master alloys in aluminum alloy melt. Figure 14(Ia,b) show TEM images of the flaky θ′ precipitate phase of uninoculated and inoculated Al-Cu alloys, respectively. As shown in Figure 14(Ia), in non-inoculated Al-Cu alloys, the θ′ phase has a thicker width that is between 10 and 15 nm and a larger average diameter, 167.7 nm; after inoculation, the θ′ phase has a larger number, a narrower width that is between 4 and 8 nm, and a tinier average size, 69.7 nm. When the content of grain boundary length increases, the distribution of CuAl_2_ phase on the grain boundary becomes more uniform, which has some influence on the precipitation kinetics. In Al-Cu alloys, the increase in grain boundaries favors the formation of defects, while the vacancy clusters formed in Al-Cu alloys may be nucleation points of precipitated phases [40]. Because there is a greater θ′ phase content and fewer solute atoms, the length of the θ′ phase is limited.

Al-Si alloys, renowned for their exceptional castability, wear resistance, corrosion resistance, and favorable mechanical properties, coupled with a low coefficient of thermal expansion, are of significant importance in various industrial applications. These alloys are widely employed in the automotive, aerospace, electronics, and machinery sectors, where their combination of performance attributes makes them indispensable for critical components. Wu et al. [82] transformed hypoeutectic Al-10Mg_2_Si alloys from coarse flake to fine coral by the addition of 0.5–3.5% Cu in Al-10Mg_2_Si alloy. The average length of the eutectic Mg_2_Si phase decreased from 15.7 μm and 2.3 μm to 6.5 and 0.4 μm with the increase in Cu content. Clearly, the addition of Cu into Al-10Mg_2_Si alloy can effectively refine the primary α-Al grain in the alloy. At the same time, the coarse crystalline Mg_2_Si grain also changed, forming a fine coral structure. The growth of eutectic Mg_2_Si crystals was inhibited by the undercooling at the grain boundary and the large number of nano-precipitated layers, which resulted in the branching and refining of eutectic Mg_2_Si crystals. (Figure 14II,III).

Based on this principle, the microstructure of aluminum alloys can be effectively tailored by controlling the morphology and distribution of the secondary phase, thereby enhancing their subsequent mechanical properties. However, to date, there remains a lack of efficient refiners capable of modulating the secondary phase in aluminum alloys both domestically and internationally. Current strategies predominantly rely on refining the secondary phase and optimizing the cooling rate to achieve microstructural control, yet these approaches often fall short of delivering consistent and scalable improvements.

## 4. In Situ Crystallization Formation of Nanocrystalline Structures: Cast Aluminum Alloy Microstructure Control Mechanism and Regulation

Cast Al-Si alloys consist of α-Al, primary (eutectic) Si, and the second phases. The size, morphology, and aspect ratio of α-Al, secondary phases, and eutectic Si critically influence the microstructural characteristics of the related alloys [117,118]. In contrast to conventional crystalline alloys, amorphous alloys exhibit long-range atomic disorder, which confers unique properties. These include high strength, enabling resistance to impact deformation; exceptional hardness, facilitating wear resistance and prolonged service life; and potential utility as catalysts, attracting significant research interest. When heated above their crystallization temperature, amorphous alloys undergo crystallization, forming nanocrystalline structures with small, uniform grain sizes and superior thermal stability. This transformation property has been leveraged by researchers to refine the microstructure of aluminum alloys.

### 4.1. Formation Mechanism of In Situ Nanocrystals

There are several interrelated mechanisms, including nucleation, growth, and competition, for the formation of nanocrystals by in situ crystallization. In the nucleation stage, energy fluctuation is one of the key inducements of nucleation in amorphous materials. An amorphous material in long-range disorder but short-range order has an inhomogeneous distribution of atomic energy. When the energy accumulated by local atomic clusters is enough to overcome the nucleation barrier, the atoms begin to spontaneously change to an arrangement pattern with crystal structure characteristics to form an initial crystal nucleus. Structure fluctuation is also very important. Amorphous materials have various atomic clusters and short-range ordered structures. Through the migration and recombination of atoms under thermal activation or other external stimulation, some of the structures can evolve into crystalline nuclei with crystallographic symmetry. In addition, compositional fluctuation plays an important role in the formation of a crystal nucleus. Because the internal compositions of amorphous materials are not uniform, the interatomic interaction and chemical environment are more favorable for meeting the compositional conditions for the formation of crystals, thus promoting the formation of a core in these regions.

Once the nucleus is formed, the growth mechanism initiates rapidly. Atomic diffusion, one of the core driving forces of crystal growth, is temperature-dependent. Atoms in the amorphous region overcome the energy barrier and migrate to the nucleus surface via thermal diffusion. The nucleus is continuously supplied with atoms, leading to an increase in its size. Interfacial migration and atomic diffusion act synergistically. During the continuous embedding of the nucleus, the interface between the amorphous nucleus and amorphous matrix moves toward the nucleus, gradually expanding the space occupied by the nucleus, and promotes sustained crystal growth. The competition mechanism that runs through the whole process of in situ crystallization plays a key role in controlling the final morphology and structure of nanocrystals. On the one hand, there is fierce competition among crystal cores. Because of the limited diffusion range of atoms, neighboring nuclei restrict each other when absorbing atoms. Cores that can absorb atoms efficiently near the source of atoms grow rapidly, while those that are far away from the source of atoms or have a weak ability to absorb atoms grow slowly or even stagnate. Some cores may be engulfed by surrounding crystals. This competitive situation guarantees the relative uniformity of nanocrystalline size. On the other hand, there is subtle competition and balance between the crystallization phase and the amorphous phase. With the gradual deepening of crystallization, the crystalline phase consumes more and more atoms in the amorphous phase, resulting in a decrease in the volume of the amorphous phase. Accordingly, the diffusivity of atoms in the amorphous phase will change with the development of crystallization, which in turn affects the growth rate of the nucleus and the final result of crystallization. In many complex alloy systems, a composite structure of nanocrystalline and amorphous phases exists, which shows the complexity of microstructure evolution.

### 4.2. Regularity and Mechanism of Amorphous In Situ Nanocrystals Regulating Alpha-Al and Si Phases

In situ nanocrystals provide ready-made nuclei for the α-Al and Si phases, reducing the energy barrier for nucleation. According to the classical nucleation theory, it is necessary to overcome the change in surface energy and volume free energy, and the existence of a nanocrystalline structure is equivalent to providing part of the formed nucleus in advance, which makes it easier for α-Al and Si phase atoms to gather and form stable nucleation on the surface, thus increasing the nucleation rate and refining the grain. At the same time, there is an interface between the nanocrystals and the surrounding amorphous matrix. This interface, as an atomic diffusion channel, accelerates diffusion and migration of α-Al and Si atoms, allowing them to reach the crystal surface faster and participate in nucleus growth. In addition, the interface can constrain the growth of α-Al and Si phase grains, limiting their growth direction and size and making the grains finer and more uniform. Bai et al. [119] reported that nanocrystals such as Fe_2_B, ZrCu (B2), and NiTi (B2) are the core of heterogeneous nucleation during liquid–solid phase transition, weakening dendrites and refining grains. At the same time, other nanocrystals not trapped by the solid–liquid interface are squeezed out of the dendrite region, and more nanocrystals are adsorbed on the solid–liquid interface, preventing the growth of particles. The results showed that the effective inoculation time is significantly longer than that of traditional inoculants (about 30 min). In fact, the effective inoculation sequence of these metal glass inoculants is Ni_60_Nb_25_Ti_15_ > Fe_79.58_B_11.16_Si_9.26_ > Zr_55_Cu_30_Al_10_Ni_5_ due to Fe_2_B and NiTi (B2) having a higher melting point than Zr_2_Cu and ZrCu (B2).

The refinement of the α-Al grain and the heterogeneous nucleation of nanocrystalline greatly affect the precipitation of the second phase in the alloy. Firstly, when the α-Al grain is refined, the grain boundary is bent more, the segregation of elements in the alloy is weakened, and the distribution of the second phase in the alloy is more uniform. During the solution treatment, the reduced atomic diffusion distance enhances solute element uniformity, while in subsequent aging treatment, the activation energy for precipitation is diminished. Additionally, the nucleation and growth of the θ’ phase and Mg_2_Si are facilitated, thereby reducing the consumption of Cu and Si atoms required for their growth and leading to refined θ’ and Mg_2_Si precipitates.

### 4.3. Manufacturing Technology for the Formation of Nanocrystals by In Situ Crystallization 

The manufacturing technology for forming nanocrystals through amorphous crystallization has experimental procedures that share commonalities while also having detailed differences due to variations in aluminum alloy types and amorphous materials. For Al-Cu alloys, when FeBSi metallic glass is used as the inoculant, first, metallic glass ribbons with the composition Fe_79.58_B_11.16_Si_9.26_ need to be prepared, and their amorphous characteristics and thermal properties are characterized by XRD and DSC. The Al-Cu alloy is melted in a graphite crucible at 1023 K, after which FeBSi metallic glass ribbons with different contents (0.05 wt.%, 0.1 wt.%, etc.) are added. After stirring, the mixture is held for different times (1 min, 5 min, etc.) and then poured into a steel mold of specific dimensions for forming. The samples are then subjected to T6 heat treatment, including solution treatment at 811 K for 12 h and aging at 438 K for 10 h. Finally, tensile and other performance tests are conducted. Meanwhile, a rapid cooling experiment is performed to simulate the crystallization process of the metallic glass in the melt, and the microstructure is analyzed using XRD, TEM, and other methods [45]. For hypereutectic Al-Si alloys, when Zr_55_Cu_30_Al_10_Ni_5_ amorphous alloy (Zr-AA) is used, the amorphous ribbons are first crushed into powder, mixed with pure Al powder at a mass ratio of 7:3 by ball milling, and cold-pressed into cylindrical samples as regulators. The Al-Si alloy is melted at 1123 K, and 0.5 wt.% of Al-P master alloy is added, stirred for 2 min, and held for 40 min. Then, different contents of Zr-AA are added, stirred for 1 min, and poured. The samples are subjected to T6 heat treatment (solution treatment at 773 K for 8 h + aging at 453 K for 6 h), and the structure and performance are analyzed through various characterization methods [46]. In Al-Cu_4_/Al-Mg_1_ alloys, using Fe_80_B_11_Si_9_ (at.%) metallic glass, a rapid cooling experiment is first conducted to determine the crystalline phases. The metallic glass fragments are ball-milled with pure Al powder at a mass ratio of 3:7 at 50 rpm for 8 h and cold-pressed into blocks of specific dimensions. The Al-Cu_4_ and Al-Mg_1_ alloys are melted at 1053 K, and after the metallic glass is added, mechanical stirring and ultrasonic stirring are performed for 2 min each. After slag removal, the melt is poured, followed by T6 heat treatment (solution treatment at 783 K for 10 h + aging at 438 K for 10 h), and related performance tests and microstructure analyses are carried out [47].

In general, these experimental procedures all revolve around the crystallization of amorphous materials in aluminum alloy melts. By controlling parameters such as the amount of amorphous addition and holding time, combined with heat treatment and various characterization methods, the influence of nanocrystals formed by amorphous crystallization on the microstructure and performance of different aluminum alloys is explored. Only specific material ratios, temperature parameters, and processing details are adjusted according to the type of aluminum alloy and amorphous material.

## 5. Solidification Microstructure, Strengthening, and Toughening of Aluminum Alloy Manipulated by Nanocrystalline Structures Formed by In Situ Amorphous Crystallization

### 5.1. Solidification Microstructure of Aluminum Alloy Manipulated by Nanocrystalline Structures Formed by In Situ Amorphous Crystallization

In order to increase the number of nucleation cores and refine the grain size during solidification, specific in situ-formed seeds, elements, or particles can promote heterogeneous nucleation. Zhu et al. [120] prepared in situ nanoparticle-reinforced Al-Si-Mg alloy by the casting method. The results showed that the coarse α-Al and Mg_2_Si in the inoculated Al-Si-Mg alloy can be effectively refined by adding 0.10 wt% refiner. The average size of eutectic silicon decreased from 26.56 μm to 11.89 μm with the increase in the amount of amorphous refiner. Nanometer Fe_2_B phase precipitation in amorphous alloys has an important effect on the grain refinement. During solidification, the Fe_2_B phase is the nucleation point of the α-Al phase, while other phases are dispersed along the solid–liquid interface and pinned by particles. With the increase in the content of the FeBSi amorphous refiner, the refining limit is reached. Therefore, a good refining effect can be obtained by selecting an appropriate refiner.

Secondly, in situ crystallization control can change the phase composition of aluminum alloy during solidification by adjusting alloy composition and the solidification process. For example, in Al-Cu alloy, controlling Cu content and adding a proper amount of an amorphous refiner allow the formation of Al_2_Cu and other strengthening phases in the solidification process. The strength and hardness of aluminum alloy can be improved significantly by the formation of these strengthening phases. Bai et al. [45] found that the improvement of properties of Al-Cu alloy by amorphous inoculation is mainly due to the refinement of α-Al grains and the formation of fine Al_2_Cu precipitates and nanometer AlCuFeMn phases. In addition, in situ crystallization control can make the reinforced phase or the second phase achieve a more uniform and dispersed distribution in the aluminum alloy matrix. For example, in Al-Mg-Si alloys, the Mg_2_Si phase is dispersed uniformly in the α-Al matrix by suitable heat treatment [120]. In addition, by means of controlling the cooling rate in the solidification process and applying an external force field (such as a magnetic field, an electric field, etc.), the growth direction and distribution state of the phase can also be changed to make the phase distribution more uniform and reasonable, thus improving the comprehensive performance of aluminum alloy.

### 5.2. Mechanical Properties and Strengthening Mechanism of Inoculated Aluminum Alloy Manipulated by Nanocrystalline Structures Formed by In Situ Amorphous Crystallization

The in situ crystallization can improve the mechanical properties of the solidified structure of aluminum alloy, including its strength, hardness, and toughness. There are two mechanisms for strength enhancement. First, in situ crystallization control can significantly refine the grains in the solidified structure of aluminum alloy. According to the Hall–Petch relationship, the smaller the grain size and the larger the grain boundary area, the greater the degree of impediment to dislocation motion and the higher the strength of the material [121]. For example, the TYS and UTS of aluminum alloys can be significantly improved by in situ synthesis of nanoparticles as heterogeneous nucleation nuclei, which allows grain size transformation from coarse columnar grain to fine equiaxed grain. On the other hand, some fine dispersed phase particles may be formed during in situ crystallization. These particles can hinder the movement of dislocations and increase the strength of materials. Similarly, the increase in resilience has two factors. Small grain size can change the propagation direction of cracks, increase the path length of crack propagation, consume more energy, and improve the toughness of the material. At the same time, uniform grain distribution can also avoid stress concentration and reduce the possibility of crack initiation. Second, in situ crystallization control is helpful for improving the uniformity of solidification structure and reducing composition segregation and microstructure defects. For example, by in situ crystallization methods, such as ultrasonic treatment, alloy elements can be more uniformly distributed in the melt, so as to obtain a homogeneous structure after solidification. Table 3 displays the comprehensive properties of some typical amorphous aluminum alloys controlled by nanocrystalline formation. Table 3 presents a comprehensive performance comparison of common typical amorphous aluminum alloys controlled by nanocrystalline formation, covering Al-Cu, Al-Si, Al-Mg, and Al-Si-Mg alloys. In the Al-Cu alloy, Zr_55_Cu_30_Al_10_Ni_5_ has high strength and good plasticity, FeBSi has relatively good plasticity, Ni_60_Nb_25_Ti_15_ has outstanding plasticity, and Al_84_Ni_10_La_6_ has acceptable strength but lacks strain data. In the Al-Si alloy, the strength and plasticity of Zr_55_Cu_30_Al_10_Ni_5_ are lower than those of the same composition in Al-Cu, FeBSi has certain performance, and NiNbTi has relatively good plasticity. The FeBSi in the Al-Mg alloy has low strength but excellent plasticity. The FeBSi in the Al-Si-Mg alloy has a certain balance between strength and plasticity. To determine which material is better, the application requirements must be considered. If high strength and good plasticity are needed, Zr_55_Cu_30_Al_10_Ni_5_ in the Al-Cu alloy is suitable; if plasticity is emphasized, FeBSi in the Al-Mg alloy performs well despite its low strength; NiNbTi in the Al-Si alloy and FeBSi in the Al-Si-Mg alloy also have their own characteristics, and materials should be selected according to actual scenarios and requirements.

In addition to the above room-temperature mechanical properties, the high-temperature tensile and creep properties are also important and should be comprehensively investigated. Zhu [125] found that the tensile strength at 250 and 300 °C is 15.3% and 10.8% higher than that of both untreated aluminum alloy and Al-Si-Cu-Ni-Mg series. The results showed that the strengthening and toughening mechanism of the alloy at high temperature is related to the formation of the second phase in the matrix and the pinning grain boundary of eutectic Si. At high temperature, the alloy strength can be improved by phase precipitation, and then the short rod-shaped eutectic Si pinning grain boundary can be strengthened. In addition, the precipitated phases hinder dislocation slip and dislocation climb, and the small-size β-precipitated phases with high density play an important role in strengthening the alloy. The increase in creep resistance is due to nanocrystalline bonding. In nanocrystalline-formation-controlled alloys, the short rod-shaped eutectic Si distributed uniformly along grain boundaries also has the effect of pinning grain boundaries, effectively hindering grain boundary migration. Finally, the high-density dislocations and smaller precipitates are controlled, which can improve the high-temperature strength and the threshold stress of high-temperature creep and reduce the creep rate.

### 5.3. Fatigue Behavior of Inoculated Aluminum Alloy Manipulated by Nanocrystalline Structures Formed by In Situ Amorphous Crystallization

**Figure 15:**
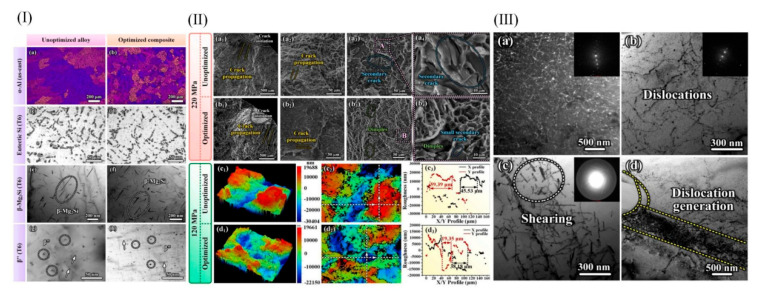
(**I**) Unmodified AlSi_7_Mg_0.3_ alloy and modified AlSi_7_Mg_0.3_ composite materials: (**a**,**b**) as-cast polarizing microscope photographs; (**c**,**d**) microscopic microscopy in the T6 state; (**e**,**f**) “β” phase observed for [001] Al Mg_2_Si after mechanical fatigue at 240 MPa or lower; (**g**,**h**) transmission electron microscopy image of [001] Al. (**II**) Observation of fatigue crack initiation points in AlSi_7_Mg_0.3_ alloy at 60 Hz using scanning electron microscopy (SEM): (**a1**–**a4**) unoptimized alloy under 220 MPa; (**b1**–**b4**) optimal composite material under 220 MPa; (**c1**–**c3**) unoptimized alloy under 120 MPa; (**d1**–**d3**) optimal composite material under 120 MPa. (**III**) Transmission electron microscopy (TEM) observations under 20 Hz and 240 MPa: (**a**) unoptimized alloys; (**b**–**d**) optimized composites: (**a**) dislocations’ dark-field image; (**b**) dislocations’ bright-field image; (**c**) interactions of dislocations with β-Mg_2_Si phases; (**d**) dislocations presented at the eutectic silicon’s corners [121].

Fatigue strength refers to the maximum stress that can be endured by materials, parts, and structures under cyclic loading without failure for a specified number of cycles. Fatigue strength is a key index in many engineering fields, such as mechanical design, aerospace, automobile manufacturing, etc. It is related to the service life and reliability of parts and structures. Fatigue resistance under extreme stress is an important index for testing the industrial application of Al-Si-Mg alloy. Liu et al. [121] found that after NiNbTi amorphous alloy crystallized into a nanocrystalline structure, the fatigue properties and microstructure of hypoeutectic Al-Si composites were obviously improved. It was found that NiTi (B2) crystals exhibited inhomogeneous nucleation and inhibition of silicon growth. Under the conditions of 120 MPa (60 Hz) and 240 MPa (20 Hz), the fatigue life is increased by about 5 times and more than 6 times compared to that of unoptimized materials. The fatigue strength at 60 Hz (N = 107) climbed by 21.7%, and that at 20 Hz by 25.9%. In an alloy, there are many key factors that affect the fatigue properties. In situ nanocrystals refined the eutectic Si phase, α-Al grain, and precipitated phase and changed the fracture morphology. The optimized fracture had smaller crack sources, fewer crack sources, narrower fatigue bands, and more small dimples. Figure 15I,II show the microstructure of an unoptimized alloy and the optimum composition in the casting and T6 states. The fracture morphology of unoptimized alloys and optimized composites at 60 Hz and 220 MPA stress amplitude is shown in Figure 15(IIa1–a4,b1–b4). This is because NiTi nanocrystals provide heterogeneous nuclei for α-Al and Mg-Si, hindering the growth of eutectic Si. NiTi phase austenite–martensite transformation provides dislocation, promotes nucleation, and refines microstructure. Moreover, refined α-Al grains make it possible to resist fatigue crack initiation, refined and spheroidized eutectic Si reduces stress concentration, and refined precipitates impede dislocation slip through the dislocation shear effect to improve fatigue strength. Figure 15III shows TEM images of unoptimized alloys and composites after mechanical fatigue at 240 MPa and 20 Hz. In addition, controlling the initial dislocation reduction of fatigue specimens in alloys improves microstructure stability. α-Al grain refinement and recrystallization grains make the fatigue crack propagation path more zigzag, and many grain boundaries make crack propagation difficult and reduce the fatigue crack propagation rate. Furthermore, the β and β” phases at the crack tip hinder the dislocation climbing, make dislocation movement difficult, and improve the high-temperature fatigue strength of the alloy.

## 6. Summary and Prospects

Aluminum alloys, with their light weight, high strength, good processability, and excellent plasticity derived from their face-centered cubic structure, are widely used in military, automotive, aerospace, electronics, and other fields [2,4]. The microstructural properties of cast aluminum alloys are closely related to the morphology, the size of α-Al, secondary phases, and the aspect ratio of eutectic silicon. This paper focuses on the research progress of regulating aluminum alloys through in situ nanocrystals formed by amorphous crystallization. Based on thermodynamic and kinetic principles, this technology can promote the formation of nanocrystalline nuclei and inhibit grain growth by adding nucleating agents and controlling the cooling rate. Thermodynamically, undercooling provides a driving force for crystallization, causing atoms to transition from disorder to order. Although nanocrystals have high interfacial energy due to their high specific surface area, their special structure and properties can reduce the total free energy of the system [21]. Kinetically, atomic diffusion is the key to crystal growth, and nanocrystals can be formed within a limited time by precise control of temperature and time [34].

At present, refiners such as Al-B, Al-Ti-C, and Al-Ti-B are widely used in industry [74]. Nanoparticles, as nucleation particles, can achieve grain strengthening. However, for Al-Si alloys with a Si content of more than 4 wt.%, their refining effect is significantly reduced. Commonly used eutectic silicon modifiers such as sodium, strontium, and rare earth elements can improve the morphology of eutectic silicon and reduce sharp corners, while the morphology and size of the secondary phase are directly related to the microstructure and application of the alloy. Compared with traditional methods such as adding refiners, in situ crystallization technology can directly induce a large number of nanocrystalline nuclei during the solidification of aluminum alloys, achieving deep grain refinement from the micron to the nanometer scale and significantly improving strength and toughness. This technology can achieve uniform distribution in the melt, ensure the consistency of grain arrangement, solve the problem of local unevenness caused by mechanical stirring, and improve material stability [119]. By refining the secondary phase and optimizing its morphology, the strength, hardness, and heat resistance of aluminum alloys can be further improved. Meanwhile, this technology has good compatibility with traditional casting and forging processes, and the melting and solidification parameters can be adjusted without major modifications to existing equipment. Moreover, it does not require complex chemical reagents, which is in line with the concepts of environmental protection and sustainable development.

It is hoped that this study can provide new ideas for the refinement of different aluminum alloys and help develop new strengthening technologies to meet the needs of the new era.

## Figures and Tables

**Table 1:** Equiaxed grain area ratio and grain size of high-strength aluminum alloy inoculated differently prepared by L-PBF method [12].

Inoculant	Composition (wt%)	Area Ratio of Equiaxed Grain Area (%)	Size of Equiaxed Grain (µm)	Content (wt%)	Inoculation Modes	Ref.
Sc	AlMg_6.66_Sc_0.66_Mn_0.51_	60.9	2.01	0.66	ball milling with ScH3 nanoparticles	[13]
	AlMn_4.69_Sc_0.7_	0	-	0.7	pre-alloyed	[14]
Zr	AlMn_4.88_Sc_1.48_	44	0.83	1.48	pre-alloyed	[14]
	AlCu_4.43_Mg_1.31_Zr_1.31_Mn_0.76_	68	0.79	1.31	pre-alloyed	[15]
	AlMg_4.13_Zr_1.42_	48.6	0.38	1.42	pre-alloyed	[16]
	AlCu_4.4_Mg_1.51_Mn_1.15_Zr_3.72_	100	0.7	3.72	pre-alloyed	[17]
Sc + Zr	AlFe_2_Cu_1.35_Zr_4.44_	100	0.785	4.44	pre-alloyed	[18]
	AlMg_5.35_Sc_0.58_Zr_0.17_	58	0.6	0.75	pre-alloyed	[19]
	AlMg_4.94_Sc_0.6_Zr_0.2_Fe_0.25_	32.6	0.68	0.8	pre-alloyed	[20]
	AlMg_6.64_Sc_0.67_Mn_0.50_Zr_0.33_	96.4	1.21	1	ball milling with ScH3 and ZrH3 nanoparticles	[13]
	AlMn_4.52_Mg_1.32_Sc_0.79_Zr_0.74_	60	0.65	1.53	pre-alloyed	[21]
	AlMg_6.64_Sc_1.07_Zr_0.54_Mn_0.50_	97.8	1.07	1.57	ball milling with ScH3 and ZrH3 nanoparticles	[13]
Ti	AlMn_5_Mg_1.5_Sc_0.82_Zr_0.76_Fe_0.16_	47.4	0.68	1.58	pre-alloyed	[20]
	AlCu_4.5_Mg_2.4_Si_1.9_Ti_0.6_	67	0.93	0.6	pre-alloyed	[22]
	AlCu_3.77_Mg_1.08_Ti_0.7_Mn_0.4_Fe_0.13_	100	2	0.7	vibrative agitation mixing with Ti nanoparticles	[23]
Nb	AlCu_2.25_Mg_1.8_Ti_1.5_	100	1.64	1.5	pre-alloyed	[24]
	AlTi_2_	100	0.522	2	-	[25]
	AlZn_12.03_Mg_2.25_Cu_1.64_Nb_1.47_	100	0.8	1.47	pre-alloyed	[26]

**Table 2:** Modification of eutectic Si by different metamorphic elements [78].

Decomposed Elements	Si Morphology	Advantages	Disadvantages
Na	Fine fibrous	Short gestation period	Easily evaporates and quickly fades away
		Insensitive to cooling rate	Low recovery rate, difficult to add and control
			Redistribution of pores
			Easily undergoes deterioration
Sr	Fine fibrous	Does not easily undergo deterioration	Long gestation period
		Lasting effect, easy to add, and good recyclability	Relatively high cooling rate
			Redistribution of pores
Sb	Layered thin slices	After remelting, it still maintains durability and metamorphic effects	Harmful gases are produced during the adding and remelting process
			Only achieved a fine layered structure

**Table 3:** Comparison of the comprehensive properties of some common typical amorphous aluminum alloys controlled by nanocrystalline formation.

Alloy	Metallic Glass	wt.%	TYS (Mpa)	UTS (Mpa)	Strain(%)	Refs.
Al-Cu	Zr_55_Cu_30_Al_10_Ni_5_	0.2	424	569	11.1	[46]
	FeBSi	0.1	-	520	14.2	[45]
	Ni_60_Nb_25_Ti_15_	0.05	363.2	534.3	19.2	[122]
	Al_84_Ni_10_La_6_	0.4	374	520	-	[123]
Al-Si	Zr_55_Cu_30_Al_10_Ni_5_	0.2	-	362	2.21	[1]
	FeBSi	0.1	-	460	5.09	[123]
	NiNbTi	0.05	228	348	18.5	[124]
Al-Mg	FeBSi	0.05	91.2	145.5	29.8	[47]
Al-Si-Mg	FeBSi	0.1	226	343	18.4	[120]

## Data Availability

No new data were created or analyzed in this study. Data sharing is not applicable to this article.
